# Marks of the True Physician

**Published:** 1877-10-20

**Authors:** 


Marks of the True Physician.—The true physician is quiet and unpretending, yet firm, prompt and attentive. He is kind and courteous in deportment, especially in the sick-room. He is jealous and careful of his reputation, but does not seek to establish it by unprofessional or unfair means, and is guarded and respectful toward the opinions and character of professional brethren. He is temperate, and should be Christian man—ready, after exhausting his skill and resources for the relief of physical suffering, to administer a balm of hope and comfort to the despairing spirit. He should be an observing man —studious, watchful, and progressive, and should read, contribute to, and *pay for* at least one medical journal. W.

